# Treating RBM20-related cardiomyopathy—Ready for prime time?

**DOI:** 10.1016/j.omtn.2025.102775

**Published:** 2025-11-26

**Authors:** Johanna Kohn, Anna-Maria Lauerer, Philipp Hegner, Simon Lebek

**Affiliations:** 1 Department of Internal Medicine II, University Hospital Regensburg, 93053 Regensburg, Germany

## Main text

Cardiovascular diseases are the leading cause of morbidity and mortality worldwide, highlighting the strong need for developing innovative therapeutic approaches.^1^ In a recent publication in *Molecular Therapy Nucleic Acids*, Roman et al. developed an advanced CRISPR-Cas prime editing (PE) strategy to correct the RBM20^P633L^ mutation, a deleterious variant that causes severe dilated cardiomyopathy (DCM). After treating human induced pluripotent stem cell-derived cardiomyocytes (iPSC-CMs), the authors observed a restoration of downstream molecular defects.^2^ These findings represent convincing evidence that PE can effectively correct pathogenic mutations associated with cardiovascular diseases.

DCM is characterized by an impaired contractile function with ventricular dilatation and is one of the most common causes of non-ischemic heart failure and trigger for arrhythmias. The pathophysiology of DCM is multifactorial. Besides acquired triggers, genetic factors play the central role in DCM development, with mutations in key target genes, such as *Titin* (*TTN*), *Lamin A/C* (*LMNA*), and *RNA-binding motif protein 20* (*RBM20*).^3^ RBM20 is an important splicing factor in striated muscle that controls alternative splicing of several cardiac genes, including those encoding sarcomeric and Ca^2+^-regulatory proteins like Ca^2+^/calmodulin-dependent protein kinase II delta (CaMKIIδ).^4^ CaMKIIδ is a major regulator in cardiac physiology and signaling. Upon sustained overactivation, it causes cardiac dysfunction and triggers arrythmias by disrupting cellular Na^+^ and Ca^2+^ homeostasis.^5^

Over the past years, CRISPR-Cas technology has evolved into a powerful tool for genome editing to treat human pathologies. PE is an advanced generation gene editing tool that enables all 12 possible nucleotide modifications as well as precise insertions or deletions. This is performed without a double-stranded DNA break by using a fusion of Cas nickase and reverse transcriptase that is directed by a prime editing-guide RNA (pegRNA). The pegRNA consists of a single-guide RNA (sgRNA), a Cas nickase scaffold, a primer binding site, and a reverse transcription template. The reverse transcription template can be designed for all types of edits.^6^ PE strategies are constantly being refined to overcome the existing limitations of established systems concerning precision and editing efficiency. The latest strategy is PE7, which demonstrates improved stability of the pegRNA component by the usage of a La domain (a small RNA-binding exonuclease protection factor), resulting in a significantly higher editing efficiency.^7^

Roman and colleagues have chosen three clinically relevant DCM mutations. LMNA^K117fs^ frameshift mutation (c.348_349insG) leads to a premature stop codon, caused by a guanine insertion in the first exon of the *LMNA* gene.^2^ RBM20^P633L^ (c.C1898T) and RBM20^R634Q^ (c.G1901A) are two missense variants resulting in toxic stress granules in the cytoplasm and incorrect splicing of major cardiac genes like *CAMK2D* (encodes CaMKIIδ).

The authors generated a target-array-HEK293T cell line to test thoroughly the gene editing efficiencies of various pegRNAs designed for each mutation. The *AAVS1* safe harbor locus was chosen to achieve stable genomic integration of the mutant gene variants. *AAVS1* (also known as the *PPP1R12C* locus) on human chromosome 19 is a well-validated safe harbor for hosting DNA transgenes with expected function as it offers an open chromatin structure and is transcription-competent. The best acting (e)pegRNA for every mutation identified in this screening platform was then transferred to human iPSC-CMs. After 20 days of differentiation, they were transduced with a recombinant adeno-associated virus (AAV) vector containing the (e)pegRNA and the components of the PE4 system. Notably, the group achieved a T-to-C editing efficiency of 34.8% for the RBM20^P633L^ mutation, which is a remarkably high efficiency giving the complexity of PE technology. Furthermore, phenotypical analyses with confocal microscopy showed that nuclear RBM20 expression and physiological splicing of *CAMK2D* were largely restored. No comparable editing efficiencies were achieved for the LMNA^K117fs^ (1.1%) and RBM20^R634Q^ (4.6%) mutations, which illustrates that achieving high PE efficiency remains complex, is locus-dependent, and that high editing efficiencies detected in one model (e.g., HEK293T cells) are not necessarily transferable to other models (e.g., human iPSC-CMs or even *in vivo*).

Previous studies demonstrated the enormous potential of PE, where the gene editing components were applied in human iPSCs before differentiation. Nishiyama et al. used a PE3bmax system combined with an (e)pegRNA to correct the RBM20^R636S^ (c.C1906A) mutation. They reached an A-to-C editing efficiency of 40% and showed a restored nuclear localization of RBM20.^4^ Chemello and colleagues used a PE3 strategy in human iPSCs, where exon 51 of the *DMD* gene was deleted. They inserted two nucleotides into exon 52 of *DMD* to correct the open reading frame and restore dystrophin expression.^8^

**Figure 1:**
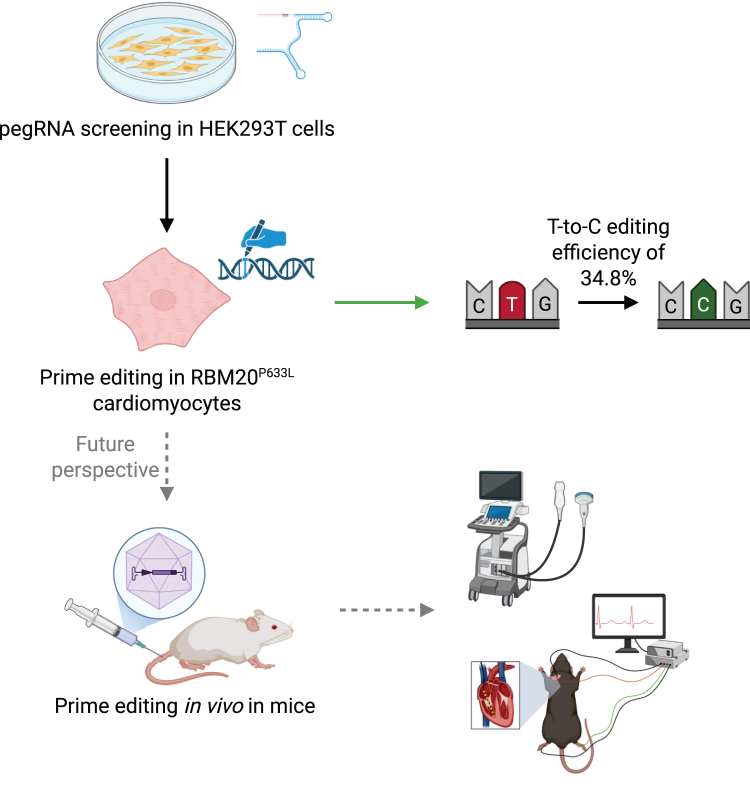
Prime editing as a therapy for RBM20-related cardiomyopathy After a thorough screening and several optimization steps, Roman and colleagues developed an advanced prime editing strategy to correct the P633L mutation in RBM20. The authors achieved a high T-to-C correction efficiency of 34.8% in mutant human stem cell-derived cardiomyocytes, and a restoration of downstream molecular defects. Future studies *in vivo* with RBM20 mouse models will be an important step toward clinical translation.

Roman and colleagues now present an excellent study with substantial methodological advances by successfully deploying AAV-delivered PE in differentiated iPSC-CMs. Several steps will need to be taken prior to potential clinical translation. Delivering the PE components *in vivo* into the heart will be a challenge as many vectors end up in the liver. In this study, the authors utilized an AAV vector, which is commonly used in the gene therapy field. Even though AAVs may induce adverse immunological side effects, they demonstrated a good transduction efficiency in the mouse heart.^6^ In addition, the recent development of myotropic AAVs enables substantial reductions in viral dose. Delivering the PE *in vivo* can effectively be tested with suitable mouse models harboring the respective mutations.^4^^,^^9^ Besides evaluating the PE editing efficiency *in vivo*, studying RBM20 mouse models allows testing whether clinically relevant endpoints like cardiac contractility (echocardiography) and arrhythmias (e.g., with heart catheterization for electrophysiological analyses) can be prevented by correcting the mutation (Figure 1).

One key question is what level of editing efficiency is required to achieve a therapeutic benefit. Most likely, correcting 100% of the mutant alleles is not required as Roman and colleagues demonstrated convincing phenotypical rescue after 34.8% correction, which is in accordance with previous studies in the gene editing field.^2^^,^^5^^,^^6^ However, the degree of correction required may also depend on the specific mutation. The R634Q mutation is one of the most pathogenic variants of RBM20 and is associated with a particularly severe clinic. Kornienko et al. described that R634Q results in almost complete cytoplasmic mislocalization of RBM20, whereas with P633L a significant fraction of the protein remains correctly localized in the nucleus. The reason for these phenotypical differences is the severe disruption of the RS domain of RBM20 induced by the R634Q mutation. This results in profound structural changes of the protein, which is difficult to reverse by only a partial correction and may require a substantial higher gene editing efficiency.^10^ Fortunately, our field is rapidly moving and constantly developing new and improved gene editing tools. When the authors started their study, they used the most recently published prime editor version at that time. Since then, improved prime editor versions with potentially higher editing efficiencies were published, raising hope for the development of effective strategies to correct other, previously difficult-to-edit mutations.

Roman and colleagues reported a conceptually important and exciting work demonstrating one of the first PE strategies in post-mitotic cells for the treatment of hereditary cardiac disease. This raises the prospect of broader applicability of this cutting-edge technology to additional mutations. Future studies evaluating these findings *in vivo* in RBM20 mouse models will be essential to confirm their therapeutic potential and may serve as a steppingstone toward clinical translation.

## Acknowledgments

P.H. is funded by a research grant from the 10.13039/501100005971German Heart Foundation (Deutsche Herzstiftung e.V) and the 10.13039/501100001659German Research Foundation (DFG; HE 10029/1-1, project number: 554804344). S.L. is funded by the 10.13039/501100001659DFG Heisenberg-Professorship (LE 5009/2-1, project number: 528296867), 10.13039/501100001659DFG research grants (LE 5009/3-1, project number: 528297116; LE 5009/5-1, project number: 554804344), a Rise up! research grant from the 10.13039/501100008454Boehringer Ingelheim Foundation, and a research grant from the 10.13039/501100005971German Heart Foundation (Deutsche Herzstiftung e.V.). Figures were created using https://BioRender.com.

## Declaration of interests

The authors declare no competing interests.
